# Predictors of Inconsistent Condom Use among a Hard to Reach Population of Young Women with Multiple Sexual Partners in Peri-Urban South Africa

**DOI:** 10.1371/journal.pone.0051998

**Published:** 2012-12-20

**Authors:** Yanga Z. Zembe, Loraine Townsend, Anna Thorson, Anna Mia Ekström

**Affiliations:** 1 Health Systems Research Unit, Medical Research Council of South Africa, Cape Town, South Africa; 2 Division for Global Health (IHCAR), Public Health Sciences, Karolinska Institutet, and Department of Infectious Diseases, Karolinska University Hospital, Stockholm, Sweden; University of Ottawa, Canada

## Abstract

**Background:**

Evidence suggests that multiple concurrent sexual partnering may be a key driver of the high HIV prevalence among young women in South Africa. However, little is known about whether and to what extent women who have multiple sexual partners also engage in other high risk sexual behaviors such as inconsistent condom use. And yet, multiple concurrent sexual partnering is of little epidemiological relevance if all partners in these sexual networks use condoms consistently. This study assesses the prevalence of sexual risk behaviors and HIV, and predictors of inconsistent condom use among women aged 16–24 with multiple sexual partners in a peri-urban setting in South Africa.

**Methods:**

We used Respondent Driven Sampling, a sampling strategy for hard-to-reach populations to recruit 259 women aged 16–24 in a bio-behavioral cross-sectional survey in the Western Cape province. Estimates of population proportions and 95% confidence intervals (CIs) were calculated using the Respondent-Driven Sampling Analysis Tool 5.6 (RDSAT). The primary outcome was inconsistent condom use in the past three months.

**Results:**

Young women reported an average of 7 partners in the past 3 months and a high prevalence of sexual risk behaviors: concurrency (87%), transactional sex (91%) and age mixing (59%). Having >5 sexual partners in the last 3 months doubled the risk of unprotected sex (OR 2.43, CI 1.39–4.25). HIV prevalence was 4% among 16–19 year olds, increasing threefold (12%) at age 20–24.

**Discussion:**

Multiple sexual partnering, where a high number of partners are acquired in a short space of time, is a fertile context for unprotected and risky sexual behavior. The young women featured in this survey present with a constellation of high-risk sexual behaviors that cluster to form a risk syndrome. Carefully tailored repeat bio-behavioral surveillance surveys are recommended for this sub-population.

## Introduction

The HIV epidemic has stabilized in South Africa, but at an unacceptably high national prevalence rate of 11% [1] with some sub-populations continuing to be more at risk of contracting HIV than others. Young women aged 15–24 are one such sub-population, as they account for 90% of all new HIV infections in their age group [2]. Further, HIV prevalence among young women increases dramatically with age: 7% among 15–19 year olds compared to 21% among 20–24 year olds and 33% among 25–29 year-olds [1]. This three- to four-fold increase in HIV prevalence among young women as they grow older demonstrates their escalating vulnerability to HIV. Owing to these HIV transmission dynamics among women aged 20–29, they have been classified as a most at risk population (MARP) [1]. Further, HIV prevalence amongst females aged 15–24 years is three to four times higher than among males of similar age [1]. Clearly, there are sustained age and sex variations in HIV transmission among young people in the country.

Structural factors such as women’s socio-economic status and gender disempowerment contribute to women’s heightened vulnerability to HIV in South Africa [3]–[5]. In addition, studies in the region point to the high prevalence of sexual risk behaviors among young women, such as early sexual debut, transactional sex, age mixing and low to no condom use [6]–[9] to explain women’s vulnerability to HIV. However, none of these studies have been conducted among women who have multiple sexual partners; a risk behavior considered to heighten the risk of HIV in the region.

Multiple sexual partnering increases the likelihood of concurrency because the more sexual partners one acquires in a short space of time, the greater the likelihood of partner overlap [10], [11]. Although not incontrovertible [12], [13], evidence suggests that concurrency is a key driver of the epidemic in sub-Saharan Africa. In high HIV prevalence settings, the density of concurrent sexual networks increases the chance that an uninfected sexual partner will encounter an acutely HIV infected sexual partner (with high viral load and greater infectivity) during an unprotected sexual act in the sexual network [10], [14]–[16].

While an association between concurrency and low condom use has been found amongst women aged 18–50 in the USA [15], it is not known whether and to what extent women who have multiple sexual partners in Sub-Saharan Africa also engage in other risk behaviors such as transactional sex (where sex is exchanged for cash or material goods), age mixing (where young women have much older male sexual partners) or inconsistent condom use. And yet, multiple sexual partnering is of little epidemiological relevance for the spread of HIV infections if all partners in these sexual networks use condoms consistently.

We aimed to assess the prevalence of sexual risk behaviors and HIV and the predictors of inconsistent condom use among young women with MSP in a peri-urban setting in South Africa.

## Methods

### Ethics Statement

Informed consent for biological and behavioral data was sought from all eligible participants. Due to the sensitivity of our research questions, parental consent for participants who were younger than 18 was not sought. Instead parent information sessions conducted by the first author were held in all the schools in the study community two months prior to the study. No parent raised concerns or indicated they would refuse for their underage children to participate in the study. The Research Ethics Committee of the Faculty of Health Sciences, University of Cape Town, granted ethics approval for the study.

### Study Setting

The study was conducted in the Western Cape province of South Africa. The Western Cape province has the lowest prevalence of HIV nationally (4%) and also among youth aged 15–24 (3%) [1]. It is also known to be one of the wealthiest provinces in the country. Our study community is a poor, Black, peri-urban township located in the Cape Winelands region of the province, approximately 60kms from Cape Town. The community has a population of 25600 people, high rates of unemployment (38%) and informal housing (75%) [17]. Formative research revealed a number of risk factors for HIV in this community; the normative practice of concurrent sexual partnering among young women, very few recreational facilities, and a large number of alcohol serving venues (n = 16) known as shebeens, that are popular among young people, and men and women who want to meet new sexual partners.

### Sampling

Women reporting multiple sexual partnerships are underrepresented in conventional surveys [15] owing to the stigmatization of this risk behavior. We used respondent driven sampling (RDS), a chain referral sampling methodology, known to be effective for the recruitment of populations that are hard-to-reach [18]–[20] to recruit women who self reported more than one male sexual partner in the past three months into a bio-behavioral cross sectional survey.

Similar to snowball sampling, RDS recruits individuals through networks of friends. However, in addition, RDS limits the influence of recruiters on the final composition of the sample (by restricting the number of recruits per recruiter); determines the personal network size of each participant (i.e. the number of peers known by each participant who represent with the population characteristics of interest); and weights the sample (by network size) to compensate for the over- or under-sampling of individuals with certain socio-demographic characteristics, thereby adjusting for the non-random sampling of participants in the analysis [20]–[22].

Due to the lack of prevalence data on condom use and related sexual risk behaviors among women who have multiple sexual partners in the region and in the country, the main outcome variable (inconsistent condom use) could not be used to calculate sample size. An approximate sample size of 270 was thus calculated based on an estimated antenatal HIV prevalence of 9% in the region, with a precision of +/−5%, and a design effect of 1.5.

### Eligibility

Eligible participants were female; aged 16–24 years; residing/working/socializing in the study community; self–reporting more than one male sexual partner in the past three months; and reporting a social network size of 1 or more women who have multiple sexual partners.

### Recruitment

Recruitment began with five initial respondents known as ‘seeds’ who reported multiple sexual partnering during informal conversations initiated by the first author at local shebeens two months prior to the study. When the study commenced in October 2007, the seeds were invited to participate in the survey at a fixed study site. Seeds were screened for eligibility and if eligible, provided informed, written consent. They were assigned a unique recruitment number to link them to future recruits and enrolled in the survey. They received three recruitment coupons with which to recruit friends and/or acquaintances. The recruitment script asked seeds (and every subsequent participant cum recruiter) to recruit friends/peers that they knew to behave in the same way as them. Cosmetics shopping vouchers were issued for completing the survey questionnaire (R30∼$4), for providing a Dried Blood Spot sample (R30∼$4) and for each successfully enrolled recruit (R20∼$2.50). This process was followed through a number of recruitment waves for every subsequent recruit who came to the study site and who successfully completed the survey.

We recruited 309 young women with a maximum of 12 recruitment waves. Fifty women (19%) were ineligible: 25 were younger or older than the age limit; 22 did not know any women who had multiple sexual partners; two did not have more than one sexual partner in the past three months and one did not stay nor socialize in the study community. The final sample was thus 259 women.

### Data Collection

Following informed consent, participants completed a pilot tested, paper based and self-administered demographic and behavioral questionnaire consisting of 103 items. The questionnaire was offered in English and in the primary, isiXhosa language of the participants. We enquired about participants` demographics such as age and poverty status. To measure levels of poverty we used a five-item Poverty Scale [23] that we then categorized to infer three levels of poorness: abjectly poor, poor and non-poor. Sexual behavior was assessed by questions about age at sexual debut; number of sexual partners; condom use in the past three months with main (non-secret, steady and predominant) and casual (secret but regular and sometimes long term) partners; condom use at last sex with once off partners (partners with whom sex was once and never again); transactional sex; age mixing; and concurrency. These variables are defined in greater detail in Annex S1.

Participants took between 30 and 60 minutes to complete the questionnaire. Three trained, Xhosa speaking young female interviewers were made available to assist participants in cases where they needed the questionnaire to be explained.

A qualified nurse collected Dried Blood Spot (DBS) samples from consenting participants immediately after the survey questionnaire was completed. All participants were offered free voluntary pre and post-test counseling and testing (VCT) for HIV at the study site. All (N = 259) consented to provide a DBS sample and 65% went for VCT.

### Data Analysis

Estimates of population proportions and 95% confidence intervals (CIs) for demographics and risk behaviors were calculated using the Respondent-Driven Sampling Analysis Tool version 5.6 (RDSAT) (Table 1). This software package enables analyses of equilibrium, and generates sample weights to take into account differential recruitment (homophily) and variations in participants’ network sizes [19].

**Table 1:** Sample & population estimates of demographic characteristics and sexual risk behaviors.

Variable	N	Sample Proportions %	Estimated Population Proportions % (95% CI)^1,2^
Age			
16–19	66	25	26 (19.0–32.0)
20–24	193	75	74 (68.0–81.0)
School Status			
Out of School	66	25	26 (16.9–27.8)
In School	193	75	74 (72.2–83.1)
Poverty Status			
Abjectly Poor	51	20	21 (16.9–27.2)
Poor	131	51	53 (45.3–58.8)
Non-Poor	72	29	26 (20.1–32.2)
HIV Status			
Negative	244	6	5 (2.7–08.4)
Positive	6	94	95 (91.6–97.3)
Sexual Debut			
≥15 years	174	60	66 (55.2–67.9)
<15 years	84	40	34 (32.1–44.8)
Condom use: main partner			
Consistent	50	19	23 (17.3–27.6)
Inconsistent	209	81	77 (72.4–82.7)
Condom use: casual partner			
Consistent	115	45	44 (37.9–50.4)
Inconsistent	138	55	56 (49.6–62.1)
Condom use: once off partner			
Yes	145	71	70 (68.7–82.9)
No	59	29	30 (17.1–31.3)
Age Mixing			
No	105	45	41 (38.4–52.3)
Yes	135	55	59 (55.2–47.7)
Concurrency past 3 months			
No	36	15	13 (12.3–23.0)
Yes	211	85	87 (77.0–87.7)
Main Partner Infidelity			
No	56	31	28 (23.4–38.9)
Yes	124	69	72 (61.1–76.6)
Transactional Sex			
No	19	7	9 (3.4–8.8)
Yes	240	93	91 (91.2–96.6)
Number of casual partners past 3 months			
≤4 casual partners	157	59	61 (52.2–64.7)
≥5 casual partners	102	41	39 (35.3–47.8)

1 Only the estimated proportions are used to describe the results in the main text.

2 Estimated population proportions were weighted using Respondent Driven Sampling Analysis Tool (RDSAT) 5.6.

A sample attains equilibrium when the sample distribution on key variables remains stable even as new individuals are added to the sample [19], [20], [21]. Equilibrium was reached between 2 and 3 waves on key variables such as age, poverty status, HIV status, inconsistent condom use with main and casual partners, male partner infidelity, transactional sex, concurrency and age mixing.

Homophily index values that are large (1+) suggest that recruiters had a greater preference for in-group recruitment; small index values (−1) indicate preference for out-group recruitment, whilst index values close to 0 suggest neither in-group, nor out-group recruitment patterns, thereby overcoming recruitment biases [20], [21]. In this study the homophily index (Hx) values for key variables ranged from −0.396 to 0.186. Hx by HIV status revealed that cross recruitment occurred among networks of HIV positive (Hx = 0.093) and HIV negative (Hx = 0.08) participants.

To determine the personal network size of each participant, we asked the following: “How many women do you know (i.e. you know their name and they know yours); you have seen them in the past three months; they are 16–24 years old; they live/work/socialize in the study community and they have had sex with more than one man in the past three months?” The personal network sizes of the participants ranged from 2 to 80 with a mean of 6.

Bivariate analyses were performed with inconsistent and no condom use as the main outcomes. To assess the predictors of inconsistent and no condom use, multivariate logistic regression models (Table 2) were constructed in STATA 10.10 using backward stepwise regression methods [24]. Individualized weights generated on the outcome variables by RDSAT 5.6 were imported and included in each of the final logistic regression models.

**Table 2:** Multivariate logistic regression models showing predictors of inconsistent condom use and no condom use.

Predictor Variables	Inconsistent Condom Use: Casual Partners OR (95% CI)	Inconsistent Condom Use:Main Partners OR (95% CI)	No Condom Use with Once-Off Partner
Age			
20–24	1.00	***	1.00
16–19	1.50 (0.82–2.74)		4.37 (1.67–11.81)
Sexual Debut			
≥15 years	1.00	***	1.00
<15 years	1.75 (0.95–3.23)		1.90 (0.91–3.94)
Age Mixing			
No	***	1.00	1.00
Yes		1.58 (0.80–3.11)	1.70 (0.82–3.78)
Concurrency past 3 months			
No	1.00	1.00	***
Yes	2.14 (0.95–4.83)	2.24 (0.95–5.28)	
Casual partners past 3 months			
≤4 casual partners	1.00	1.00	
≥5 casual partners	2.43 (1.39–4.25)	2.22 (1.07–4.60)	***
Access to condoms at shebeens			
No	***	1.00	1.00
Yes		0.46 (0.20–1.07)	0.45 (0.20–0.97)

*** Predictor variables where p>0.25 in the bivariate analyses were excluded from the multivariate logistic regression model.

We also conducted Mantel-Haenszel tests for homogeneity to determine if transactional sex modifies the interaction between inconsistent condom use with casual partners and concurrency.

The DBS were analyzed at a referral laboratory for anonymous HIV testing where serum was eluted from samples and tested and re-tested with a 4th generation HIV ELISA (Vironostika Uniform II plus 0) and a 3rd generation (antibody only) HIV ELISA (SD Bioline). Samples that were reactive in both assays were reported as positive. Discordant samples were tested by western blot (HIV1/2 Biorad).

## Results

All young women reported sexual relations with a main partner, an average of 7 male sexual partners in the past three months and a very high proportion (87%, CI 77.0–87.7) reported concurrency within these sexual relationships. Table 1 shows the prevalence of sexual risk behaviors. Despite the fact that 73% (CI 67.6–78.4) reported easy access to condoms at shebeens, the majority reported inconsistent condom use both with their most recent main partner (77%, CI 72.4–82.7) and casual sexual partners (56%, CI 49.6–62.1) in the past three months. Among those who reported having sex with once off partners in the past three months (82%, CI 64.3–95.9), 30% (CI 17.1–31.3) did not use a condom at last sex with their most recent once off partner.

More than 9 out of 10 young women (91%, CI 91.2–96.6) reported that they had engaged in transactional sex with their most recent sexual partner. More than one third (34%, CI 31.2–36.7) reported early sexual debut (<15 years old), 59% (CI 55.2–47.7) reported age mixing and 72%, (CI 61.1–76.6) knew or suspected their main partner to have other sexual partnerships (male partner infidelity). Most of the respondents were aged 16–19 (74%, CI 68.0–81.0), with 21% (CI 16.9–27.2) reporting abject poverty.

Women who reported a high number of multiple casual sexual partners (five or more casual partners in the past three months), were also significantly more likely to have unsafe sex (inconsistent condom use) both with their casual partners (OR 2.43, CI 1.39–4.25), and with their most recent main partner (OR 2.22, CI 1.07–4.60), compared to those with fewer partners (Table 2). Transactional sex did not modify the interaction between inconsistent condom use with casual partners and concurrency (crude OR 1.93, CI 0.94–3.95; adjusted OR 1.93, CI 0.94–3.96).

The youngest women (aged 16–19) were more likely to not have used a condom at last sex with the last once-off partner (OR 4.37, CI 1.67–11.41) compared to older women (aged 20–24). Early sexual debut (<15 years) was also marginally associated with the likelihood of not using condoms at last sex with this type of partner (OR 1.90, CI 0.91–3.94). Women reporting easy access to condoms at shebeens were 55% less likely to not have used condoms at last sex with their most recent once off partner (OR 0.45, CI 0.20–0.97).

The prevalence of HIV was three times higher among women aged 20–24 (12%; CI 5.3–22.0) than among women aged 16–19 (4%, CI 1.4–7.3). Average HIV prevalence across age groups was 5% (CI 4.1–6.5).

## Discussion

This is the first study in South Africa to be conducted among a heterosexual, young female subpopulation who self-report having multiple sexual partners. In this township sub-population, we found a strikingly high prevalence of high-risk behaviors for HIV. Despite the fact that the majority were still in school and less than a quarter reported abject poverty, most young women had engaged in transactional sex, and practiced unsafe sex with concurrent, considerably older partners, many of whom also had other sexual partners.

In a context where strict monogamy is considered the exception rather than the rule [25]–[27] and where material rewards are generally considered a common feature of successful sexual relationships [25], [27], [28] classifying women who have multiple sexual partners and comparing them to other groups in South Africa is complex. First, despite the high number of partners and the high prevalence of transactional sex among them, this subpopulation cannot be simply classified as commercial sex workers (CSWs) [3], [28], [29]. Two studies that have measured sexual risk among commercial, female sex workers in Pretoria [30] and in Johannesburg [31], report dynamics of risk that may be similar to what we found among our subpopulation: a similarly high prevalence of inconsistent condom use with main partners (59%) in spite of almost half (41%) of the population of CSWs reporting male partner infidelity [30] and levels of condom use with clients (85%) [31], comparable to what was reported for condom use at last sex with once off partners in our study. However, and importantly, despite these similarities, the terms and conditions of the sexual exchange are organized differently in the two groups, resulting in different opportunities for safer sex negotiations. Among young women who have multiple sexual partners, the exchange of monetary rewards occurs in the context of romantic relations, often after sexual intercourse has taken place and relies on implicit and symbolic rather than explicit communications about the type and price of the sex act [3], [27], [32], [33]. The absence of an empowering context that allows for explicit and upfront negotiations about the type and cost of sexual encounters prior to the sex act means that young women who are in transactional sexual relationships of this kind are constrained in their ability to proactively determine the sexual [safety] terms of the sexual act [29], [32]. This is perhaps one area where CSWs, who negotiate sex for exchange encounters preliminarily and explicitly, are positioned advantageously to women who engage in transactional sex. These differences are important to emphasize if appropriate and tailored intervention strategies are to be developed for women who engage in transactional sexual relationships, particularly as this risk behavior is more common in peri-urban settings than commercial sex work [27].

Clearly the practice of transactional sex is not untouched by the prevailing gender norms that undermine women’s sexual and reproductive health in the country [27]. However, transactional sex should not be simply reduced to a behavior where women are construed as mere passive victims who have no opportunities for agency and enactments of empowerment in their relationships [30], [31], [41], [4]. An important question regarding displays of agency is whether they translate to empowerment that is sexually protective and meaningful for HIV risk reduction among young women [34].

Further, the high prevalence of transactional sex in a context where more than two thirds of the population are classified as poor and non-poor rather than abjectly poor, suggests that the popularity of the risk behavior may have more to do with wealth inequalities than abject poverty [28], [35].

Together, these findings emphasize the need for a more nuanced understanding of transactional sex; one that clearly distinguishes transactional sex from commercial sex work, leading to interventions that are better equipped to deal with the complexity of this risk behavior and its driving factors.

The high number of sexual partners observed among the young women may be indicative of the multiplicity of young women’s needs [25], which may not be sufficiently met by main, single partnerships. This proposition is particularly compelling in light of emerging evidence that young women’s main partnerships in South Africa may be socially structured to be “traditional” [34], with emphasis on enactments of masculinities and femininities that encourage male partners to be dominant, controlling, and emotionally reserved, whilst requiring women to be sexually timid, submissive and tolerant of their main partners’ misbehavior. Thus young women may be acquiring various sexual partners, to meet multiple needs, be it love, sexual pleasure, material rewards or insurance against abandonment by their current main sexual partner [25], [28]. Interventions that target this subpopulation need to take into account the ways in which social beliefs about men and women’s roles in their intimate steady relationships configure the size and function of their multiple partnerships.

Together with the high levels of inconsistent condom use, multiple sexual partnering and concurrency, the reported high prevalence of male partner infidelity may lead to very densely populated sexual networks, otherwise known as “concurrency superhighways” [17], which facilitate a wider spread of HIV [16]. Further, the highly significant association between inconsistent condom use and a high number of partners in a short period of time suggests that multiple sexual partnering creates a fertile environment for unsafe sexual practices. The finding also highlights the importance of interventions that emphasize partner reduction among populations where multiple sexual partnering is a norm.

The high prevalence of age mixing among our study participants could explain the low use of condoms. Older men often take on a dominant position in age-asymmetrical relationships [7]–[9] and are known to use their age (and economically) related power to undermine young women’s preferences for safer sex [5], [7]. This is particularly concerning in a context where the prevalence of HIV has been shown to increase drastically among older men, suggesting that when young women establish unprotected sexual contacts with this group they are essentially entering and mixing with a high HIV seroprevalence pool [1], [2], [36]. Thus, interventions that reduce the prevalence of age mixing could significantly reduce the prevalence of HIV among young women [37].

Notably, women who reported easy access to condoms in alcohol serving venues were more likely to use condoms with once off partners. This is an indication of the large gains to be made if condom availability is extended to spaces where young people socialize and where transactional sexual relationships are initiated.

The drastic increase in HIV infections among women aged 20–24 compared to those aged 16–19 in our sample mirrors the trend observed in national household survey data, which show a relatively low prevalence of HIV for women under 20 years of age (7% among 15 - 19 year olds), but a three-fold increase among women aged 20–24 (21%) [1]. These findings highlight that much can be done to prevent HIV in this subpopulation if interventions target women earlier, specifically when they are young adolescents. Further, the overall HIV prevalence of 5%, whilst almost double the provincial prevalence of 3% among youth aged 15–24 [1], supports emerging evidence that suggests that high-risk behaviors do not necessarily translate to a high prevalence of HIV in low prevalence settings [38].

We deliberately recruited women who self-reported multiple sexual partnering because of the strong hypothesized link between this behavior and HIV prevalence and gaps in knowledge about this and concomitant risk behaviors in the Sub Saharan African region. While multiple sexual partnering is common among women in the general population [25], [10], current national household surveys provide limited knowledge about this high-risk behavior. As such, what we know about women who have multiple sexual partners is based on knowledge derived from qualitative studies but rarely confirmed through quantitative enquiries. Thus, whilst conclusions about the extent to which our subpopulation is representative of all women who have multiple sexual partners should be made cautiously, these results make an important contribution to our understanding of this risk group. They show not only that these women have high numbers of partners, but that they also engage in a range of other high risk behaviors such as transactional sex and age mixing, whilst foregoing consistent condom use with their various sexual partners, making them a high risk group for HIV infection.

### Limitations

While women are known to under-report sexual behaviors [1], [11] we believe the use of a peer recruitment strategy and our efforts to ensure confidentiality and anonymity provided a greater opportunity for women to report honestly. Despite this strength, our study has limitations. First, although we set eligibility criteria that limited enrolment to women reporting more than one sexual partner in the past three months, women in the general population, such as those who have serial rather than concurrent multiple sexual partners were not precluded from participating in the study. However, the results indicate that we largely captured a high-risk subpopulation, since nearly 9 out of 10 women reported concurrency. Second, although providing important information about the extent of risk behaviors among women who have multiple sexual partners, owing to the limited scope of this study, we are not able to make sufficient conclusions about the size of this risk group in the general population. Third, the sample size was calculated to detect HIV prevalence and associated risk among women of reproductive age (15–49). However, the recruitment strategy and the eligibility criteria that we used yielded a largely adolescent age cohort (age group 16–19) that is known to have a relatively low HIV prevalence [1]. Fourth, we used a single item measure of concurrency in our study, which might be limited in light of the UNAIDS recommendation that measures focus on coital exposure, overlap duration and point prevalence [39].

### Conclusions

Four main implications emerge from these findings. First, young women who have multiple sexual partners present with a risk profile marked by a constellation of risk behaviors, clustering to form what is known as a “risk behavior syndrome” [40] which heightens their vulnerability to HIV. Thus, addressing single isolated acts, rather than the range of interlinked, sexual risk behaviors, will not be effective. Second, multiple sexual partnering creates a favorable environment for unsafe sex practices to flourish; thus interventions that target this subpopulation must emphasize consistent condom use with all sexual partners. Third, the context of gender and economic inequalities that encourages the practice of transactional sex and compromises young women’s ability to negotiate safer sex, calls for structural, socio-economic interventions that provide women with alternatives (such as conditional cash transfers that are linked to behavior change [41] and that alter the social environment within which they make decisions about their sexual practices. Creative strategies that redefine and link ideas of pleasurable, profitable sex to sexual health and safety are also relevant. To optimize the viability and success of such strategies, the full engagement of young women and men as partners in the development, implementation and evaluation of interventions that target them is necessary. Finally, given the very high prevalence of sexual risk behavior and gaps in knowledge about the magnitude of multiple sexual partnering in South Africa, repeat bio-behavioral surveillance studies that make use of survey methods that are carefully tailored to elicit accurate disclosure of multiple sexual partnering and associated high risk behaviors among sexually active young women in the general population, are recommended.

## Supporting Information

Annex S1 Definition and coding of variables.
(DOC)
