# Supplementary material for: Predictors of Inconsistent Condom Use among a Hard to Reach Population of Young Women with Multiple Sexual Partners in Peri-Urban South Africa
Source: PLoS One. 2012 Dec 20;7(12):e51998. doi: 10.1371/journal.pone.0051998 (PMC3527429; doi:10.1371/journal.pone.0051998)
Supplement: Annex S1 — Definition and coding of variables. (DOC) [file pone.0051998.s001.doc]

**Annex S1**. Definition and coding of variables

| **Variable** | **Survey questions and responses** | **Codes** | **Explanation of codes** |
| --- | --- | --- | --- |
| **Poverty Status** | Which ONE of the following best describes how things are in your home? | 1 | Abjectly poor |
|  | We do not have enough money for food | 2 | Poor |
|  | We have enough money for food but we do not | 2 | Poor |
|  | We have enough money for food and clothes but we do not have money for many other important items | 2 | Poor |
|  | We have money for all the important items but very little for luxurious items | 2 | Poor |
|  | We have enough money for luxurious items and other items | 3 | Non-poor |
| **Sexual Debut** | How old were you when you first had sex? | 0  1 | ≥ 15 years  < 15 years |
| **Condom Use Indicators** | | | |
| Condom use with main/casual sexual partner(s) past 3 months | How often have you used condoms with your [main partner or casual partner] in the last 3 months? Would you say never, sometimes, often or always? | 0  1 | Consistent (always)  Inconsistent (never, sometimes or often) |
| Condom use with once-off sexual partner(s) past 3 months | Think back to the most recent once-off sexual partner that you had sex with, the last time you had sex with him did you use a condom? | 0  1 | Yes  No |
| **Transactional sex** | Think about your most recent [main, casual, once-off sexual partner] do you think you became involved in a relationship with him because you were expecting or because he gave you any of the following: food, clothes, cosmetics, cell-phone, airtime voucher, (material items), cash? TICK AS MANY AS APPLY | 0  1 | No  Yes |
| **Concurrency** | In the last 3 months did you initiate/enter a new sexual relationship whilst in an existing and ongoing sexual relationship with another sexual partner? | 0  1 | No  Yes |
| **Age mixing** | In the last 3 months did you enter a sexual relationship with a man who was 5 or more years older than you? | 0  1 | No  Yes |
